# A high-throughput strategy for screening of bacterial artificial chromosome libraries and anchoring of clones on a genetic map constructed with single nucleotide polymorphisms

**DOI:** 10.1186/1471-2164-10-28

**Published:** 2009-01-18

**Authors:** Ming-Cheng Luo, Kenong Xu, Yaqin Ma, Karin R Deal, Charles M Nicolet, Jan Dvorak

**Affiliations:** 1 Department of Plant Sciences, University of California, Davis, CA, 95616, USA; 2 Genome Center, University of California, Davis, CA, 95616, USA

## Abstract

**Background:**

Current techniques of screening bacterial artificial chromosome (BAC) libraries for molecular markers during the construction of physical maps are slow, laborious and often assign multiple BAC contigs to a single locus on a genetic map. These limitations are the principal impediment in the construction of physical maps of large eukaryotic genomes. It is hypothesized that this impediment can be overcome by screening multidimensional pools of BAC clones using the highly parallel Illumina GoldenGate™ assay.

**Results:**

To test the efficacy of the Golden Gate assay in BAC library screening, multidimensional pools involving 302976 *Aegilops tauschii *BAC clones were genotyped for the presence/absence of specific gene sequences with multiplexed Illumina GoldenGate oligonucleotide assays previously used to place single nucleotide polymorphisms on an *Ae. tauschii *genetic map. Of 1384 allele-informative oligonucleotide assays, 87.6% successfully clustered BAC pools into those positive for a BAC clone harboring a specific gene locus and those negative for it. The location of the positive BAC clones within contigs assembled from 199190 fingerprinted *Ae. tauschii *BAC clones was used to evaluate the precision of anchoring of BAC clones and contigs on the *Ae. tauschii *genetic map. For 41 (95%) assays, positive BAC clones were neighbors in single contigs. Those contigs could be unequivocally assigned to loci on the genetic map. For two (5%) assays, positive clones were in two different contigs and the relationships of these contigs to loci on the *Ae. tauschii *genetic map were equivocal. Screening of BAC libraries with a simple five-dimensional BAC pooling strategy was evaluated and shown to allow direct detection of positive BAC clones without the need for manual deconvolution of BAC clone pools.

**Conclusion:**

The highly parallel Illumina oligonucleotide assay is shown here to be an efficient tool for screening BAC libraries and a strategy for high-throughput anchoring of BAC contigs on genetic maps during the construction of physical maps of eukaryotic genomes. In most cases, screening of BAC libraries with Illumina oligonucleotide assays results in the unequivocal relationship of BAC clones with loci on the genetic map.

## Background

In species devoid of complete genomic sequence information, libraries of bacterial artificial chromosome (BAC) clones [1] are an indispensable genomic tool. The utility of BAC libraries has been further enhanced by the development of high-information-content-fingerprinting (HICF) techniques [2-4] and the FPC program for the assembly of fingerprinted BAC clones into contigs [5-7], which opened the door to the construction of BAC-based physical maps of plant and animal genomes (for review see [8]). An operation common to virtually all applications of BAC libraries is screening the libraries for clones harboring specific nucleotide sequences. Current screening techniques utilize either DNA-DNA hybridization or polymerase chain reaction (PCR). In some applications, such as the construction of a physical map, a BAC library must be screened for the presence of hundreds or thousands of different molecular markers. To maximize the efficiency of such screening, multidimensional pooling of clones or probes is employed [9]; probes are pooled if a library is screened by DNA-DNA hybridization [10] and clones are pooled if it is screened by PCR [11].

All current screening strategies are laborious, slow, error prone, and often result in ambiguous assignments of BAC clones to loci on a genetic map. The identification of BAC clones harboring specific nucleotide sequences by hybridization of multidimensional pools of overgo probes with BAC library screening membranes [10], in addition to some of the above problems, also requires handling large amounts of radioactive material. The greatest impediment to screening of BAC libraries with pools of cDNA clones or overgo probes is that a single probe often hybridizes with clones in multiple contigs, either due to gene duplication, the presence of repeated sequences, or other reasons. Unequivocal assignment of BAC clones to loci on the genetic map requires additional work.

We describe here a BAC library screening strategy that is largely devoid of these limitations and can be performed in a high-throughput mode. The strategy employs Illumina GoldenGate™ oligonucleotide assays, also referred to as Oligonucleotide Pool Assays (OPAs), that are currently used for the highly parallel SNP genotyping of genomic DNAs [12]. Each assay targets a specific SNP locus and utilizes two allele-specific oligonucleotides to discriminate between SNP alleles. The allele discriminating nucleotide of an allele-specific oligonucleotide is at its 3' end. Another primer, the locus-specific oligonucleotide, which contains an address sequence for the SNP locus, anneals downstream of the SNP. After annealing one of the two allele-specific oligonucleotides to the genomic DNA template, the oligonucleotide is extended by DNA polymerase and ligated to the locus-specific oligonucleotide downstream forming a contiguous PCR template. Primer extension and ligation can be performed at up to 1536 loci simultaneously. The templates are PCR amplified using three PCR primers complementary to specific sequences inserted into all oligonucleotides. Two primers anneal to the allele-specific oligonucleotides; one for each SNP is labeled with the Cy3 fluorochrome and the other with the Cy5 fluorochrome. The third anneals to the locus specific oligonucleotide. The ratio of the Cy3 and Cy5 fluorescence is used to determine the genotype at a SNP locus. If the ratio is near 0 or 1 (near pure Cy3 or near pure Cy5 fluorescence), the locus is homozygous. If the ratio is about 1: 1 the locus is heterozygous.

It is shown here that annealing of allele- and locus-specific oligonucleotides to a pool of BAC DNAs and the subsequent primer extension and ligation reaction can be used to determine whether or not a BAC pool DNA harbors a specific locus. This allows genotyping the BAC pool for the presence or absence of the locus. It is also shown that BAC genotyping with Illumina oligonucleotide assays results in a high percentage of unequivocal assignments of BAC clones and BAC contigs to loci on a genetic map.

A six-dimensional BAC pooling strategy has previously been successfully used to genotype 24576 sorghum BAC clones (about 4× sorghum genome equivalents) for the presence or absence of specific amplified fragment length polymorphism (AFLP) amplicons during sorghum physical map construction [11]. The pooling strategy utilized 184 pools. Although the six-dimensional strategy worked well for the sorghum genome, it results in too many pools for large genomes, such as those of wheat and its diploid ancestors. One genome equivalent of *Aegilops tauschii *(1C = 4,020 Mb [13]), one of the three diploid ancestors of polyploid wheat, amounts to 30000 to 40000 BAC clones, depending on the average size of DNA inserts. To facilitate screening multiple genome equivalents of large genomes, such as that of *Ae. tauschii*, but keeping the numbers of pools manageably low, a simple, five-dimensional pooling strategy was designed and evaluated here.

Contigs built from 199190 *Ae. tauschii *BAC clones [14] fingerprinted with the SNaPshot HICF technology [4] were simultaneously screened with 1384 multiplexed Illumina GoldenGate assays. Assignments of BAC clones and contigs to gene loci on the *Ae. tauschii *chromosome 2D genetic map were analyzed to assess the proportion of BAC clones and contigs unequivocally assigned to individual loci on the genetic map.

## Results

### Genotyping of genomic DNA and BAC pool DNA with Illumina GoldenGate assays

**Figure 1:**
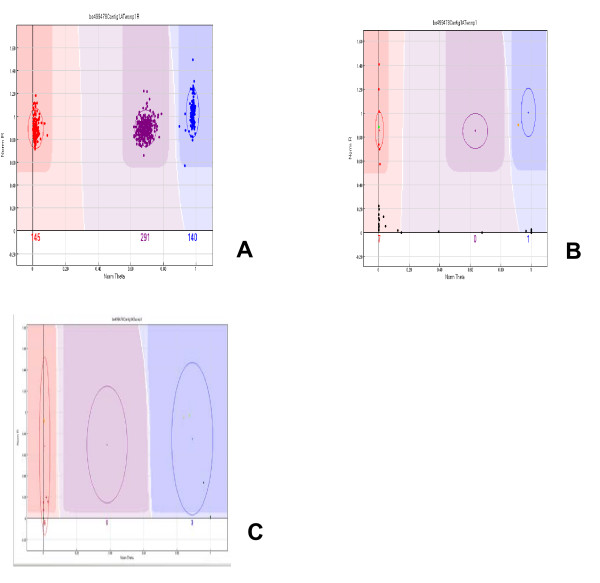
**(A) Genotyping of parental lines and 576 *Ae. tauschii *F_2 _plants in the AL8/78 × AS75 mapping population with Illumina GoldenGate oligonucleotide assay querying an A/G SNP at EST locus BE499478 (nucleotide position 403 in the *Ae. tauschii *reference sequence, )**. The graph is a normalized polar coordinate (*R*, *θ*) plot. Theta (*x*-axis) is an angle of deviation of Cy3 and Cy5 fluorescence from pure Cy3 and pure Cy5 signal (0 and 1). The closer a point is to 0 or 1, the greater the proportion of Cy3 or Cy5 fluorescent signal, respectively, is present. *R *(*y*-axis) is the Manhattan distance of observed Cy3 and Cy5 fluorescence to the origin (pole), which is 0 fluorescence. The closer a point is to 0 on the *y*-axis, the weaker the total fluorescence. The red, blue and purple ovals have the diameter of 2 standard deviations computed from the dispersal of the red, blue and purple dots, respectively. The darker colored regions define genotype call areas for homozygous (red and blue dots) and heterozygous (purple dots) plants. The numbers of plants in each cluster are indicated below the *x*-axis. (B) Clustering of 57 BAC and BiBAC super-pools assayed with the same oligonucleotides as in (A). Genotype call areas derived from the mapping population shown in 1A were directly exported for analysis of the BAC pools. The green and orange spots represent the parental genotypes of the mapping population; the red spots are the BAC and BiBAC pools scored by us as positive and black spots BAC and BiBAC pools scored as negative. Note that all red spots have the same normalized angular deviation (normalized theta) as DNA of *Ae. tauschii *AL8/78 and large distance from origin (*R *value) indicating high fluorescence. In contrast, the black spots have a low *R *value indicating only residual Cy3 and Cy5 fluorescence and variable values of normalized theta ranging from 0 all the way to 1. (C) Clustering of the same 57 BAC and BiBAC super-pools without defining call areas on the basis of clustering segregating *Ae. tauschii *SNP genotypes. Note failure of separation of the positive and negative clusters from each other.

Genotyping of BAC super-pools consisting of pooled BAC plate-pools and containing DNA of either 10368 or 11520 clones with an Illumina GoldenGate assay is illustrated in Figure 1, using oligonucleotides designed for an A/G SNP at locus BE499478. In contrast to SNP genotyping, the query in BAC genotyping is whether or not an SNP allele of *Ae. tauschii *accession AL8/78 (the source of DNA used for the construction of the BAC library) is present in DNA of a BAC super-pool. BAC super-pools containing BAC clones harboring the AL8/78 target sequence are expected to show Cy3/Cy5 fluorescence (normalized theta in Fig. 1) similar to that of plants homozygous for the AL8/78 genotype in the F_2 _population (red dots in Fig. 1A); those that do not contain such a BAC are expected to show no or residual fluorescence. Super-pool DNAs containing clones with the AL8/78 BE499478 allele (the red dots in Fig. 1B) were within the call region ("cluster") exported from the plot of the F_2 _plants genotyped with allele- and locus-oligonucleotides specific for BE499478 (Fig. 1A). BAC super-pools with a null for the targeted BE499478 sequence showed only residual Cy3 and Cy5 fluorescence, and their Manhattan distance (sum of the Cy3 plus Cy5 fluorescence) from the origin (0 in the plots) in the normalized polar coordinate plot placed them below the call area (black dots in Fig. 1B). Because the fluorescence of these DNAs was residual, their normalized theta did not cluster but ranged from 0 to 1. Without defining the call areas on the basis of the previous clustering of the F_2 _plants (Fig. 1A), it would have been impossible to separate positive and negative super-pools from each other (Fig. 1C).

### BAC clone, BAC contig and genetic locus correspondence

Oligonucleotides for 1536 *Ae. tauschii *SNPs were designed and multiplexed but only 1383 proved useful to interrogate DNA of 574 *Ae. tauschii *F_2 _plants because for 153 of them AL8/78 and AS75 parents were monomorphic or were not homozygous. Three distinct clusters (two homozygotes and the heterozygote), similar to those shown in Fig. 1A, were obtained for 1212 sets (87.6%). The 1212 SNPs were mapped at 705 EST loci (some loci contained several SNPs) on the *Ae. tauschii *map (to be reported elsewhere). Genotype call areas exported from these data were used to genotype BAC super-pools into positive and negative clusters (as shown in Fig. 1B). All Illumina assays that successfully clustered F_2 _genomic DNAs also successfully clustered super-pools into positive and negative clusters.

Contig locations of 241 BAC clones detected with a subset of 43 Illumina oligonucleotide assays were investigated (Table 1). Of these, 11 BAC clones (indicated by * in Table 1) were not present among the fingerprinted clones and were excluded from further consideration. Clones detected with 38 Illumina oligonucleotide assays were in single contigs (Table 1). Those detected with Illumina oligonucleotide assays for loci BE497494, BG263521, BQ161196, BE499671, and BE497590 were each in two contigs. Contig pairs harboring clones detected with Illumina assays for loci BE497494, BG263521 and BQ161196 could be merged into single contigs with FPC. The positive clones were neighbors in the reassembled contigs. Five clones detected with the Illumina oligonucleotide assay for marker BE499671 were in contigs ctg5065 and ctg7763 (Table 1). The two contigs could be merged only above a Sulston score of 1 × 10^-2 ^and positive clone HB012I17 of ctg7763 was separated by 10 clones from the group of positive clones present in ctg5065. Both facts suggest that the two contigs are from different parts of the genome. Hence, the Illumina assay for locus BE499671 failed to generate unambiguous contig anchoring on the genetic map. A similar anchoring failure was observed for locus BE497590, as nine BAC clones identified by this oligonucleotide assay were assembled in two contigs (ctg144 and ctg8859) that could not be merged (Table 1). Overall, in the trial of 43 markers, 95.3% of them resulted in a one-to-one relationship of clones in a contig to locus on the genetic map.

**Table 1:** Anchoring of *Ae. tauschii *contigs on the genetic map of *Ae. tauschii *chromosome 2D with Illumina GoldenGate oligonucleotide assays.

Locus	Map position (cM)	Positive clones anchored	Contig
BE489611	28.2	RI063B22, HI094B3, HB051N10	ctg9144
BF201235	28.3	HD143B20, RI053K20, RI127F20, HI043L21, HD013G23	ctg4666
BE518440	46.2	HB123G1, RI004M10, HI113P4, RI105L19*	ctg6274
BE500206	63.8	HI143K4, HI003A22, HI012B19, HI090K7, HD123H5, RI056P19, BB048O9	ctg7899
BE497494	85.9	BB093B14, HB123K12, HB008P16, HB133H13	ctg8357**
BE497494	85.9	HB004F21, HB123L18, HB123M5, HD008O15	ctg5568**
BE405367	87.3	HD087H2, HD141H13, HD137I4, HB059H9, RI112C12, RI118E2, HD121I8, HI123C14, BB100F2*	ctg10452
BE499671	88.9	HB012H8, HI129G1, HD057E7, HD121M12	ctg5065
BE499671	88.9	HB012I17	ctg7763
BE499478	88.9	HI090L10, RI035P6, BB079N19, BB110C10, HB043B9	ctg1170
BE445242	89.0	HD072M21, HB007A23, HI050N18, HB015E4, HB035C6, HB036O19, HI039G10, HB060O15, HD151B19	ctg9317
BE498730	89.0	HB030C17, HB033C8	ctg1617
BF202681	89.0	HD116C6, HB009B12, HB040H21, HI140N22, HI124O3	ctg6734
BQ169707	92.3	RI103N16	ctg1280
BF478936	92.7	HD039I3, RI012F23, HD107L14, HI077L6, RI070K18, RI024B7*	ctg8860
BE497590	96.9	RI015E2, HI117D24, HI148P10	ctg144
BE497590	96.9	HI053D12, HI037I5, HI094B9, HI122O11, HI152F13	ctg8859
BG313656	98.1	HB034O15, HB062N11, RI112D8, HI029J12, HD032F21	ctg982
BG263521	98.8	HD132P10, HB021B10, HD033G4, HI148C2, BB067M15, BB081E2, BB107K11, RI043J14 *	ctg7791**
BG263521	98.8	BB081O16, RI032D19	ctg2288**
BF291674	102.4	HD074O4, BB054A2, HB027O2, RI107N10, RI136G23, HI037F12	ctg10410
BF483083	102.4	BB038N07, HB023N15, HB081O4, HD080K19, HI078L18, HI146A6, RI084G17, BB123G5*	ctg2905
BE517627	103.1	HD065I16, HD050E11, HD053J2, HD057A20, HB055D1, HB083C24, RI076J11	ctg4985
BE442608	103.1	BB092N18, BB070C1, HB006B8, HB086J7, HB096K20, HD133P21, HI137N13, RI019F6	ctg4985
BE591248	103.3	RI020P1, RI091B2, HD091O22, HD137A8, HB078N22, BB032J4,	ctg3732
BE404384	104.2	HD062D2, BB047B24, HB110L22, HI080L19, RI061L5, RI064P6	ctg6492
BE405045	104.3	HD074J1, HD068K4, HI052G12, RI076M20, RI124J20	ctg5942
BQ161196	104.3	HB067J7	ctg8076**
BQ161196	104.3	HB125P19, HB075P15, BB003G7	ctg6731**
BG606625	104.4	BB061O1, BB061P1, HB040G1, HB082C18, HB084M10, HD026P1, HD112E21 HI152K3, RI018P5, RI131L21, RI146B11	ctg3670
BE604861	104.4	HB002K4, HD135K22, RI013H19, RI023K17, BB083K1*	ctg5648
BE490204	107.1	BB011G22, HD119G21, HI105F9, HD006K24*	ctg4240
BG313179	109.8	HD133K07	ctg3100
BE499362	109.8	HD074A1, HB001O3, HI143M21, HD034C5, RI051M5	ctg11438
BE406509	110.7	HI004N13, BB104N3, HD120D6	ctg251
BE442788	111.7	HD075A10, HD123P4, HI026J16, HI061H17, HI116C6, RI119E3	ctg4801
BF145580	111.8	HB003H1, BB025P18, HD005P3, HD049H11, HI116D20, HI130J21, RI136C15,	ctg4928
BE403597	113.5	RI083G1, RI113K18, RI143I24, HI019M23	ctg977
BG274019	117.1	HB006A23, HD052A8, HD063B18, HD066I10*	ctg1700
BE444264	118.8	HB012D18, BB092N14, HB032L12, HD051K23, HI090M22, RI107M7	ctg4732
BE406908	120.9	BB056F7, HB061D24, HB081H12, HB131E21, HD020E20, HI031B4, RI126O19	ctg3828
BF201830	121.3	HB075L11, RI069N16, HI024C6, HI027G18, RI069N16, BB136N11*	ctg5622
BQ169383	121.3	HD114P13, BB065I18, BB071O3, RI046H10, HI002O17, HI041M18	ctg5622
BF473744	135.1	BB019A18, BB097O11, HB119C7, HB123C17, RI035O12, RI062D7	ctg7871
BE517946	157.8	HI142E11, HD063P2, HI124C3, RI012J19, BB194H17*	ctg7891
BF483221	170.7	HB007M06, HD026P3, HI132A22, HD056A9	ctg1415
BE426620	179.2	HB104E22, HB117D08, RI101P15, RI112H4, HI065F13	ctg11040
BE490384	186.9	BB067N14, HB092N14, HD108D13, BB018I20*	ctg3479

* Clones not found among the fingerprinted clones used for contig assembly. ** The two successive contigs were merged with FPC.

### New five-dimensional pooling strategy

The pooling strategy employed above included 8.5× genome equivalents of BAC clones. On average, between eight and nine BAC clones are expected to harbor a target sequence in this genome coverage. If, e.g., there would be eight such clones in the super-pools and each would be present in a different 384-well plate, the clones may be in as many as eight plate rows and eight plate columns of the two dimensional grid, resulting in up to 64 row by column intersections. Of these intersections, only eight harbor positive plate-pools; the remaining are false positive. The truly positive plate-pools can only be found by additional PCR as done above. This represents a lot of work if a library is screened with hundreds or thousands of markers.

This additional work would be reduced if screening could be limited to a 1× genome equivalent, each screened separately. In the *Eco*RI BAC library, the 1× genome equivalent corresponded to about 100 384-well plates.

To test empirically the efficacy of this 1× genome pooling strategy, the 20 super-pools (10 RSPs plus 10 CSPs) of 1× genome equivalent of the *Eco*RI library previously genotyped with 14 Illumina oligonucleotide assays for loci mapped on *Ae. tauschii *chromosome 2D were employed. The RPs and CPs were screened by PCR. The position of each positive clone was compared with the neighboring clones in the contig to determine if some of the neighboring clones were also positive with the same marker during the BAC super-pool screening with Illumina oligonucleotides described above. Four (27%) markers detected a single BAC clone harboring a target sequence in the 1× genome equivalent (Table 2). The remaining ten (73%) detected either no clone or more than one clone resulting in two or more false-positive pools that had to be eliminated by an additional PCR (Table 2).

**Table 2:** Numbers of positive clones detected with Illumina oligonucleotide primers in the 1× genome equivalent of the *Eco*RI *Ae. tauschii *BAC library and the positive clones detected in contig in the remaining BAC and BiBAC libraries.

Locus	Map position (cM)	Contig	All positive clones in the contig	No. positive clones in 1× genome equivalent
BF428792	66.5	ctg11447	HD091B17, HB035D2, HB048A19, HD131L18, RI135A13, HI004M3	0
BE471274	89.2	ctg487	BB037M12, HB007I22, HB057P16, HB085A14, HI006N2, HD032D6, HI031J11	0
BE590745	101.2	ctg11005	HB132A7, RI076L24, HI039I15	0
BE591939	102.3	ctg6341	HD086O22, RI011N13, RI050D20*, RI053E15, HI059D10, HD120B16, HI105L1	1
BF201348	102.4	ctg9913	BB005K5, BB003G23, BB051C23, BB106M18, **RI074C16**, HI056I10	1
BM137697	121.4	ctg5641	BB054M2, RI041O13, RI140B8	0
BQ168191	93.0	ctg4237	HD145O11, HD088L23, RI089E13, HI030D11	0
BE471132	0.00	ctg6261	HD080D15, HD023E5, **RI005I8**, HD124N6, HI042G4, HI104G24	1
BE445628	85.9	ctg3619	RI130I10, HI112C21	0
CD452951	103.1	ctg4985	HD076J10, HD004P4, HD059D21, RI144L6	0
BE403177	109.0	ctg5209	BB014B15, BB012C19, HD087J6, RI048C1, RI090E18, HI014F23, HI146N21	2
BE399200	109.8	ctg5429	RI048D18, BB028N11, RI114F3, RI127A20	2
BE406351	113.6	ctg3299	HD085I19, HD133F6, RI064D18	0
BE444599	143.4	ctg19	HB012E19, HB084C20, **RI074P11**, HD028J11	1
BE445431	151.5	**	RI119I14, RI101I14, RI125I14	3

* Clones in bold were detected with Illumina and PCR in the 1× genome equivalent of the *Eco*RI library. ** Clones were detected but were not fingerprinted.

## Discussion

The Illumina GoldenGate oligonucleotide assay, originally developed for high-throughput genotyping of SNPs, has been successfully adapted to genotyping of radiation hybrids [15,16]. It is shown here that the assay can also be used for the genotyping of BAC multidimensional pools. While the query asked in SNP genotyping is which of the two nucleotides is at an SNP site, the query asked in BAC pool genotyping is whether a target sequence is present or absent. In spite of this query difference, the high success rate characteristic for the Illumina GoldenGate platform in genotyping SNPs was also achieved in BAC pool genotyping (87.6%).

It is shown here that BAC pool genotyping is best performed in parallel with SNP genotyping in order to generate well defined clusters for the sequences under investigation. This fact should be considered in the planning phase of a physical mapping project since BAC libraries should ideally be constructed for one of the parents of the segregating population on which the genetic map will be based.

An important asset of Illumina genotyping noted here is the one-to-one relationship between contigs and markers on the genetic map. In this study, 95.3% of markers resulted in anchoring single contigs at single loci on the genetic map. For comparison, of 127 cDNA clones used earlier as probes in hybridization with screening membranes of *Ae. tauschii *libraries , 55 (43%) detected BAC clones in single contigs and 72 (57%) detected clones in two or more contigs. Only the former clones could be considered anchored in this case. However, because only a subset of the clones was interrogated with cDNA probes, a portion of the 43% apparent successes was based on a single clone hit. By definition, this can be in only a single contig. If the entire set of five libraries was hybridized with the cDNA clones, the success rate would have been lower than 43%. Similar ambiguities were observed in anchoring soybean HICF contigs by screening multidimensional BAC library pools with SSRs [17].

The multiplexed Illumina oligonucleotide assays are most time and cost effective if large numbers of markers in large numbers of DNAs are genotyped and if no additional work is needed to identify positive BAC clones. A simple BAC pooling strategy is suggested here that groups the clones into 1× genome equivalents, each treated independently during screening with the Golden Gate assay. This strategy maximizes the likelihood of only a single positive clone present among the BAC pools and minimizes the need for an additional PCR to discriminate between positive and false positive pools if more then single positive BAC is present among the pools. The theoretical probabilities of encountering 0, 1, 2, ... *n *positive clones during screening of a 1× genome equivalent follows a Poisson distribution with μ = 1. The probability of detecting only a single clone by screening 1× genome equivalents of clones is 36.7%. If the effects of sampling are neglected, the empirical success rate is expected to be lower than the theoretical expectation because of occasional false negatives (failures to detect a clone when it is present in a pool). This was borne out here, as the empirical rate of detecting a single clone by screening 1× genome equivalent BAC clones was 27%. Based on this empirical rate, a minimum of four 1× genome equivalents should be screened to detect at least one positive clone. Four 1× genome equivalents for the *Ae. tauschii *genome represent 240 pools. Because the representation of a specific sequence in a BAC library depends on the distribution of restriction sites, it is desirable to combine 1× genome equivalents from BAC libraries generated with different restriction endonucleases, or utilize random BAC libraries.

The library screening technique described here is time effective since it takes less than two weeks to screen five hundred DNAs for 1536 loci and only several weeks to process the data. The cost of screening BAC libraries with GoldenGate assays is comparable or only slightly higher that the cost of overgo screening. However, the need for labor to resolve equivocal anchoring is greatly reduced with the GoldenGate assays. A factor that will likely limit the rate with which BAC libraries can be screened and BAC contigs anchored on a genetic map is the availability of sufficient numbers of single-copy SNP loci for OPA design. It is usually sufficient for anchoring of a contig on a genetic map to anchor a single locus per contig. Contig assembly may produce up to 4,000 to 6,000 contigs in large plant genomes, and their anchoring will place high demands on the availability of sufficient numbers of single-copy SNPs. The new DNA sequencing platforms will undoubtedly play a role in overcoming this potential rate limiting step.

## Conclusion

While it has been possible to perform BAC fingerprinting and the construction of genetic maps in high-throughput mode, BAC contig anchoring on a genetic map has resisted scaling to high-throughput methods. This limitation is overcome with the Illumina BAC library screening and contig anchoring technique described here. These complementary, high-throughput techniques open the door to efficient construction of physical maps of virtually all eukaryotic genomes.

## Methods

### BAC libraries and HICF fingerprinting

Three libraries constructed in a bacterial artificial chromosome vector (pECBAC1) and two libraries constructed in an Agrobacterium binary vector (pCLD4541) of *Ae. tauschii *accession AL8/78 [18] were used. Clones of the *Eco*RI, *Hin*dIII and *Bam*HI BAC libraries were designated RI, HD and HI, respectively, and those of the *Hin*dIII and *Bam*HI BiBAC libraries were designated HB and BB, respectively. Numbers of clones, coverage, and the average insert sizes are summarized at . The five libraries comprise a total of 302976 clones, which equal 8.5× *Ae. tauschii *genome equivalents.

A total of 256942 BAC clones were fingerprinted, BAC fingerprints were automatically edited with the computer program package GenoProfiler [19] and 199190 BACs clones were used for contig assembly [14]. BAC clones used for assembly represented approximately 5× genome coverage. The total length of contiged DNA represented ca. 85% of the genome.

### Clone pooling for the evaluation of library screening with GoldenGate assays

A total of 302976 clones were pooled. Three types of pools were generated: (1) pools of the 384 colonies arrayed within a single plate (plate-pools), (2) two-dimensional (2-D) pools of the plate-pools (super-pools), and (3) 2-D pools of colonies within a single plate (clone-pools).

(1) A plate-pool was generated by inoculating 50 ml of LB broth containing 12.5 mg/L of chloramphenicol (BAC clones) or 12.5 mg/L of tetracycline (BiBAC clones) in a plate devoid of wells (USA Scientific, cat. no. 2977-8510) with the BAC or BiBAC clones from a single 384-well plate with a 384-pin replicator. Cells were grown overnight, sedimented by centrifugation and DNA was isolated using a standard alkaline lysis protocol. A total of 789 plate-pools were produced. To save cell stocks for future applications, 200 μl of cell culture was added to 30 μl of glycerol and cells were stored frozen at -80C.

(2) To generate super-pools, the 789 plate-pool DNAs were arranged in a grid consisting of 27 rows and 30 columns. Equal amounts of DNA of the plate-pools in a row were pooled to produce a row super-pool. Likewise, equal amounts of DNA of the plate-pools in a column were pooled to produce a column super-pool. The 2-D super-pool grid consisted of 57 super-pools; 27 row super-pools (each containing 11520 clones) and 30 column super-pools (each containing 10368 clones).

(3) A total of 40 clone-pools were generated per plate; 16 row-pools and 24 column-pools. A plate containing 16 row reservoirs (Seahorse Bioresearch Company, cat. no. S30034) with 2.0 ml of 2 YT broth each and a plate containing 24 column reservoirs (Seahorse Bioresearch Company, cat. no. S30035) with 1.2 ml of 2 YT broth each, were prepared for each 384-well library plate. The two plates were inoculated with cells from a 384-well plate with a 384-pin replicator. Cells were grown for 20 h at 37C and the 40 clone-pools derived from a single 384-well plate were directly used for PCR screening to identify a well containing a positive clone.

### PCR conditions and PCR screening

Super-pools, plate-pools and clone-pools were screened with PCR to find clones harboring a specific *Ae. tauschii *DNA sequence. PCR was performed in a 20 μl reaction mix containing: 1× PCR buffer (1.5 mM MgCl2 included, Applied Biosystems, Foster City, California), 250 uM dNTPs (Promega), 0.5 unit of Taq-gold DNA polymerase (Applied Biosystems, Foster City, California), 10 pmole of forward and reverse primers, and an appropriate amount of template DNA or cells (2 ng for super-pools, 1 ng for individual plate-pools or 1 μl of clone pools). An ABI 9700 thermocycler was used to perform PCRs using the following regime: 94C for 5 min, followed by 35 cycles of 94C for 1 min, 56C for 1 min and 72C for 2 min, then hold at 72C for 7 min and finally a 4C stand-by. The presence of PCR amplicon and its size was determined by 1% agarose electrophoresis using 1× TAE buffer.

Fifty-seven *Ae. tauschii *super-pools were assayed with the Illumina GoldenGate assay. The plate-pools at the intersections of positive super-pool rows and columns were screened with PCR using conserved PCR primers  to identify plate-pools that harbored a positive clone. The positive clone within a positive plate-pool was identified by PCR; the positive clone was at the intersection of the positive row and positive column.

### Ilumina GoldenGate™ genotyping

Information about genotyping with the Ilumina GoldenGate assay can be found at the Illumina web site . The construction of the genetic map based on Illumina GoldenGate™ SNP genotypes will be described in detail elsewhere. Briefly, 1536 DNA sequences harboring SNPs between *Ae. tauschii *ssp. *strangulata *accession AL8/78 (supplied by V. Jaaska, University of Tartu, Estonia) and *Ae. tauschii *ssp. *tauschii *accession AS75 (supplied by C. Yen, The Triticeae Research Institute, Sichuan Agricultural University, China) were downloaded from the wheat SNP database  and supplied to Illumina Inc. (San Diego, California, USA) for the design of allele- and locus-specific oligonucleotides. Multiplexed oligonucleotides were used to genotype 574 *Ae. tauschii *F_2 _plants from the cross AL8/78 × AS75, and the AL8/78 and AS75 parental lines. All genotyping assays were carried out in the DNA Technologies Core of the UC Davis Genome Center.

For the current study, 16 position BeadChip™ arrays were utilized and scanned with the Bead Station 500. Data output was initially analyzed with the Beadstudio v.3.1.14 program using the default parameters. Further manual analysis was carried out to determine if the clustering of the three codominant genotypes was adequate. If it was found inadequate, that SNP was excluded from further work. Of the 1536 targeted SNPs, 153 were not present between the parents or the parents were heterozygous. These were excluded from consideration. Hence 1383 oligonucleotide sets ("SNPs") were studied in total.

To determine the effects of BAC pool DNA concentration on the detection of a positive super-pool and data clustering, the multiplexed GoldenGate assay was performed with DNAs of 3 randomly selected BAC super-pools, using 5 μl of 50, 25, 10, and 1 ng/μl super-pool DNA, and 5 μl of 50 and 25 ng/μl genomic DNAs of AL8/78 and AS75 as positive controls. The 50, 25 and 10 ng/μl super-pool DNA concentrations resulted in similar clustering of data but the 1 ng/μl super-pool DNA concentration showed variable clustering compared to the other three DNA concentrations. It was therefore concluded that BAC super-pool DNA concentrations between 10 and 50 ng/μl were suitable for Ilumina GoldenGate assays and 25 ng/μl was used throughout.

### Design of a new, five-dimensional clone pooling strategy

**Figure 2:**
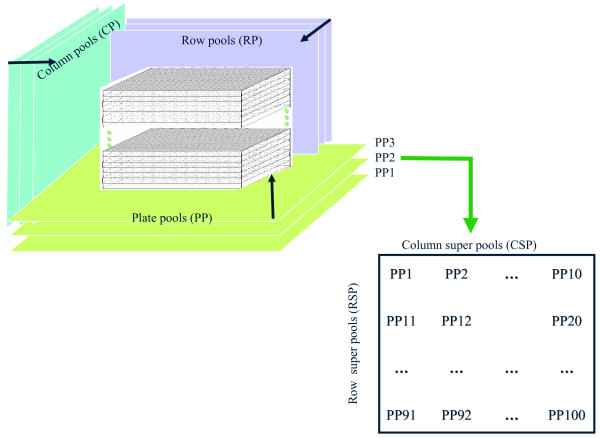
**The new five-dimensional pooling strategy**. The clones in the stack of 100 384-well plates approximately equivalent to 1× *Ae. tauschii *genome were pooled in three dimensions: row-pools (RP), column – pools (CP) and plate-pools (PP). The 100 PPs were arranged in a 10 × 10 two-dimensional grid and pooled in the row super-pools (RSP) and column super-pools (CSP).

The 384-well plates containing *Ae. tauschii *BAC or BiBAC clones were divided into groups equivalent to one *Ae. tauschii *genome. The average insert size of the five libraries was about 110 kb, and one *Ae. tauschii *genome equivalent corresponded to 36000 clones. This is equivalent to 94 384-well plates. For the sake of pooling simplicity, 100 384-well plates were used. Clones within a stack of 100 plates were pooled in the three dimensions: row-pools (RP), column-pools (CP) and plate-pools (PP) (Fig. 2). A RP was generated by pooling all clones in a specific row from the 100 plates. Each RP contained DNA of 2400 clones (24 × 100). A CP was generated by pooling all clones in a specific column from the 100 plates. Each CP contained DNA of 1600 clones (16 × 100). A PP was generated by pooling the 384 clones in a plate into a single pool. To reduce the number of PPs, the 100 plate-pools were arranged into a two-dimensional 10 × 10 grid (fourth and fifth dimension). Plate-pools in a row were pooled into a row super-pool (RSP) and plate-pools in a column were pooled into column super-pool (CSP) (Fig. 2). The total of 38400 clones contained in the 100 plates were pooled in five dimensions into 60 pools (16 RPs, 24 CPs, 10 RSPs and 10 CSPs). In the evaluation of the 5-dimensional pooling strategy, RSPs and CSPs were screened with Illumina whereas RPs and CPs were screened with PCR.

## Abbreviations

BAC: bacterial artificial chromosome; HICF: high-information-content-fingerprinting; PCR: polymerase chain reaction; OPA: Oligonucleotide Pool Assay; SNP: single nucleotide polymorphism; PP: plate-pool; SP: super-pool; CP: column-pool; RP: row-pool; CSP: column super-pool; RSP: row super-pool.

## Authors' contributions

MCL, KX and JD planed the work. MCL prepared DNAs for Illumina genotyping and performed most analyses. JD drafted the manuscript. KX constructed BAC pools, performed PCR analysis. YQM helped with PCR analysis. KRD hybridized cDNA probes with BAC library screening membranes. CN oversaw Illumina genotyping and assisted with Illumina data analysis. All authors read and approved the final draft of the manuscript.
